# A new African soft scale genus, Pseudocribrolecanium gen. nov. (Hemiptera: Coccoidea: Coccidae), erected for two species, including the citrus pest P. andersoni (Newstead) comb. nov

**DOI:** 10.1673/1536-2442(2006)6[1:ANASSG]2.0.CO;2

**Published:** 2006-03-31

**Authors:** Takumasa Kondo

**Affiliations:** Department of Entomology, University of California, 1 Shields Avenue, Davis, California 95616-8584, U.S.A

**Keywords:** coccids, new genus, taxonomic keys

## Abstract

A new African genus of soft scale insects, Pseudocribrolecanium gen. nov. is erected to accommodate Akermes colae Green & Laing and Cribrolecanium andersoni (Newstead). The adult females and first-instar nymphs of the two species are redescribed and illustrated. Taxonomic keys to separate the adult females and first-instar nymphs are provided. The affinity of Pseudocribrolecanium with the tribe Paralecaniini in the subfamily Coccinae is discussed.

## Résumé

Un nouveau genre africain de cochenille, Pseudocribrolecanium gen. nov. est créé pour les espèces Akermes colae Green & Laing et Cribrolecanium andersoni (Newstead). Les femelles adultes et les larves du premier stade des deux espèces sont redécrites et illustrées. Une clé dichotomique est proposée pour les femelles adultes ainsi qu'une clé pour les larves. L'affinité du genre Pseudocribrolecanium avec la tribu des Paralecaniini, dans la sous-famille des Coccinae, est discutée.

## Introduction

There are about 270 species in 56 genera of soft scale insects (Hemiptera: Coccoidea: Coccidae) hitherto described from the African continent (Ben-Dov et al. 2005). These have been described mainly during two periods: (i) from 1899–1941, during which more than one hundred species were named by such early workers as Newstead, Hall, Green, and Laing, and (ii) from 1954–1979 when about another hundred species were added mainly by De Lotto and Hodgson (Ben-Dov et al. 2005). Since then, various authors, including Hodgson, have sporadically contributed to the knowledge of the coccid fauna of Africa. Despite its rich flora, which includes 6 biodiversity hotspots (Myers et al. 2000; Myers 2001), the number of described coccid species recorded in the African continent has not increased in the last 10 years, undoubtedly because of the lack of taxonomic specialists in the region.

The author had the opportunity to travel to Ghana in June, 2005, and collected scale insects of various families including the Coccidae, Diaspididae, Margarodidae, Ortheziidae, Pseudococcidae and Stictococcidae. Among the coccids, a scale insect later identified as Akermes colae was collected on the leaf of a cacao tree. The genus Akermes Cockerell (1902) currently includes 14 species distributed in Central and South America (10 species), India (1 species), Australia (2 species), and Africa (1 species). The author has studied the genus Akermes previously and considers that it is endemic to the New World (Kondo & Williams 2004). Attempts were therefore made to allocate A. colae to an appropriate genus. When Green & Laing (1924) described Akermes colae, they pointed out that A. colae was structurally very similar to A. andersoni (referred to as Lecanium andersoni). De Lotto (1968) redescribed A. bruneri Cockerell, the type species of Akermes, and stated that A. andersoni had little in common with the genus Akermes and transferred it to Cribrolecanium Green (1921). However, De Lotto (1968) did not study A. colae, and it has remained in the genus Akermes until the present.

Cribrolecanium currently contains 3 species, 2 from the Oriental region and C. andersoni from the Afrotropical region (Ben-Dov et al. 2005) although the latter species has been considered only doubtfully congeneric (Hodgson 1994) based on adult female morphology. However, studies based on crawler morphology suggest that C. andersoni is definitely not congeneric (Kondo and Williams 2002; Williams and Kondo 2002). As indicated by Green & Laing (1924), C. andersoni and A. colae are closely related, but do not fit into any known genus, and thus a new genus Pseudocribrolecanium is erected here to accommodate them.

## Materials and Methods

Specimens were slide mounted according to methods discussed by Kosztarab (1996) and Kondo & Gullan (2005). Morphological terminology follows mostly that of Hodgson (1994). Measurements of specimens were made using an ocular micrometer on an Olympus phase-contrast microscope. The illustrations of the coccids follow the style adopted for the Coccoidea, with the dorsal surface drawn on the left side of the drawing and the ventral surface drawn on the right, with enlargements of important features placed around the illustration. The total number of specimens used for each description is given in parentheses, e.g. (n=48). In the material studied, the number of slides and specimens on each slide are given as the number of slides followed by the total number of specimens and their corresponding stages; for example, 1 slide with 1 second-instar female and 1 adult male specimen would be: 1(2: 1 second-instar female + 1 adult male). The stage is not given if all specimens are adult females.

### Depositories

AUCC: Auburn University Coccoidea Collection, Alabama, U.S.A.

BMNH: the Natural History Museum, London, U.K.

## Results

### Taxonomy

Pseudocribrolecanium gen. nov. keys out to the subfamily Coccinae, tribe Coccini, in the keys to subfamilies, tribes and type species of genera of Coccidae provided by Hodgson (1994). However, it does not fit well into any known genus.

### Subfamily Coccinae, Tribe Coccini Pseudocribrolecanium Kondo, new genus Type species

Akermes andersoni Newstead, 1917, by present designation.

### Generic diagnosis, adult female

Insects oval, pyriform to asymmetrical in shape, flat, becoming sclerotized at maturity.

#### Dorsum

Derm membranous, becoming highly sclerotized at maturity. Dorsal setae short, sharply spinose. Simple pores present or absent. Dorsal microducts present, rather few, scattered throughout dorsum. Translucent furrows present, associated with stigmatic areas and often elsewhere; furrows either touching or not touching margin. Dorsum usually with several cribriform plates, although occasionally absent on P. colae. Dorsal tubercles and tubular ducts absent. Preopercular pores usually present in a longitudinal line anterior to anal plates. Anal plates together quadrate or inverted pyriform, dorsal surface with 3 or 4 apical or subapical setae, plus 1 seta on inner margin of each plate. Anal ring with 4 pairs of setae and an irregular row of translucent wax pores. Eyes present, represented by clear areas located on dorsal submargin.

#### Margin

Derm heavily sclerotized in a narrow band around margins. Marginal setae numerous, slender, flagellate, arranged in a single row. Stigmatic clefts shallow or absent, with 3, rarely with fewer or up to 5 stigmatic setae; with either one seta longer than others or all subequal in length.

#### Venter

Ventral derm membranous. Ventral microducts present, small, scattered evenly on venter. Mouthparts well-developed, often displaced to one side; labium 1 segmented, with 4 pairs of setae. Pregenital disc-pores each with 5–11 loculi, mostly 10-locular; scarce, present around vulvar area and sparsely distributed on abdominal segments. Ventral tubular ducts absent. Spiracular disc-pores with 3–5 loculi, present in a single line extending laterally from each spiracle to body margin. Antennae each 2–5 segmented, segmentation indistinct; fleshy setae present only on last antennal segment and setae often knobbed. Legs greatly reduced, segmentation poorly defined; claws without a denticle. Spiracles rather small.

### First-instar nymph.

Body elongate oval.

#### Dorsum

Dorsal derm membranous. Dorsal setae short, present in 2 longitudinal parallel rows of 5 setae. A trilocular pore present on each side of head region near margin. Dorsal microducts present in a submarginal and 2 submedian longitudinal rows, with a few additional microducts on thorax between the submedian and submarginal rows. Simple pores each closely associated with a microduct. Anal plates each triangular, dorsal surface with a shingled texture and 3 apical setae, 1 seta along inner margin of each plate, with 1 fringe seta and 1 ventral subapical seta. Anal ring with 3 pairs of setae and an irregular row of translucent wax pores. Eyes located on margin on area laterad to each antennal scape.

#### Margin

Outline smooth. Marginal setae slender, total 34, with 8 anteriorly between eyes and, on each side, 3 between each eye and anterior stigmatic setae, 2 between each anterior and posterior stigmatic setae and 8 between each posterior stigmatic setae and posterior end. Stigmatic setae in groups of 3, well differentiated from marginal setae; median seta bluntly spinose, or clavate; lateral setae short, bulbous, length about 1/5–1/11 of median seta.

#### Venter

Ventral derm membranous. Pairs of submedian abdominal setae present on last 3 abdominal segments. Submarginal setae arranged in a parallel row of 7 on each side of abdomen, plus 1 submarginal seta between each anterior and posterior spiracle, and 1 submarginal pair of setae near apex of head. Interantennal setae 1 pair. With 1 ventral microduct present between each pair of submarginal setae on abdomen and 1 between each anterior and posterior spiracle. Spiracular disc-pores each with 3 loculi; 3 or 4 pores between each anterior and posterior spiracle and margin. Mouthparts normal; labium with 4 pairs of labial setae. Legs well-developed; microctenidia present on tibial apex. Prothoracic tarsal digitules dissimilar, one knobbed and other spiniform; mesothoracic and metathoracic tarsal digitules similar, both knobbed. Spiracles normal, with a well developed muscle plate. Claws each with a denticle; claw digitules knobbed, one slightly broader than other. Antennae each 6-segmented, with 3rd antennal segment longest; fleshy setae present on last 3 apical segments; 1 single fleshy seta on segment 4, 1 fleshy seta plus 1 setose seta on segment 5, and several fleshy and setose setae on terminal segment.

### Etymology

The genus name is derived from the prefix Pseudo-(Greek), meaning something superficially resembling the original subject, and the genus name Cribrolecanium Green.

**Key to species of Pseudocribrolecanium, gen. nov. based on adult females**
1. Dorsum with both large and small cribriform plates, smaller plates near margin each 10–80 μm wide, larger plates farthest from margin each 100–180 μm wide; present in about 2 or 3 irregular rows totaling 12–22 around body P. andersoni (Newstead), comb. nov.
– Dorsum with or without cribriform plates, when present, each plate small and subequal in size, 15–50 μm wide; present in 1 irregular row totaling 7–9 plates around body P. colae (Green & Laing), comb. nov.

**Key to species of Pseudocribrolecanium, gen. nov. based on first-instar nymphs**
1. Median stigmatic seta of each cleft bluntly spinose, apex round, not swollen; head rounded P. andersoni (Newstead), comb. nov.
– Median stigmatic seta of each cleft clavate, with a swollen apex; body narrowing at head P. colae (Green & Laing), comb. nov.

**Figure 1.**
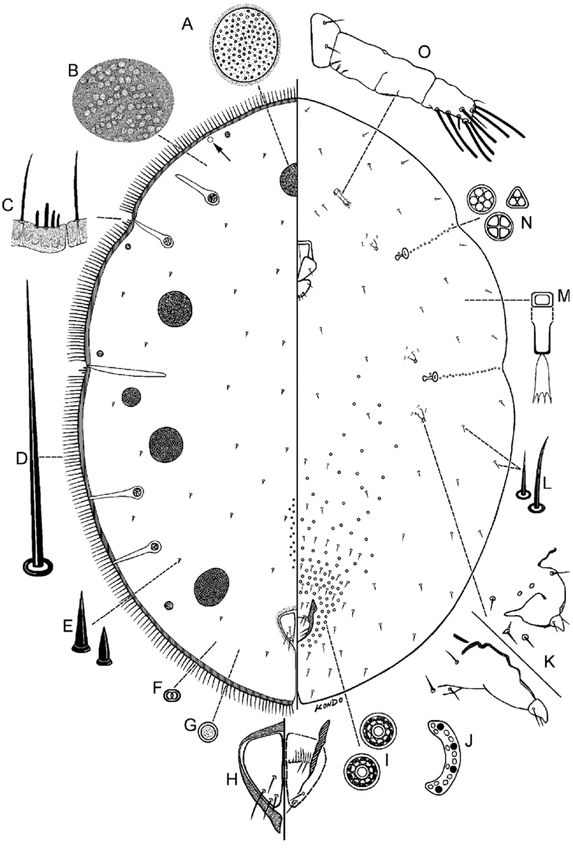
Pseudocribrolecanium andersoni (Newstead), adult female. A, cribriform plate; B, enlargement of dorsal derm; C, enlargement of sclerotized marginal band with marginal and stigmatic setae; D, marginal setae; E, dorsal setae; F, dorsal microduct; G, simple pore; H, anal plates; I, pregenital disc-pores; J, anal ring (right half); K, reduced legs; L, ventral setae; M, ventral microduct; N, spiracular disc-pores; O, antenna.

**Figure 2.**
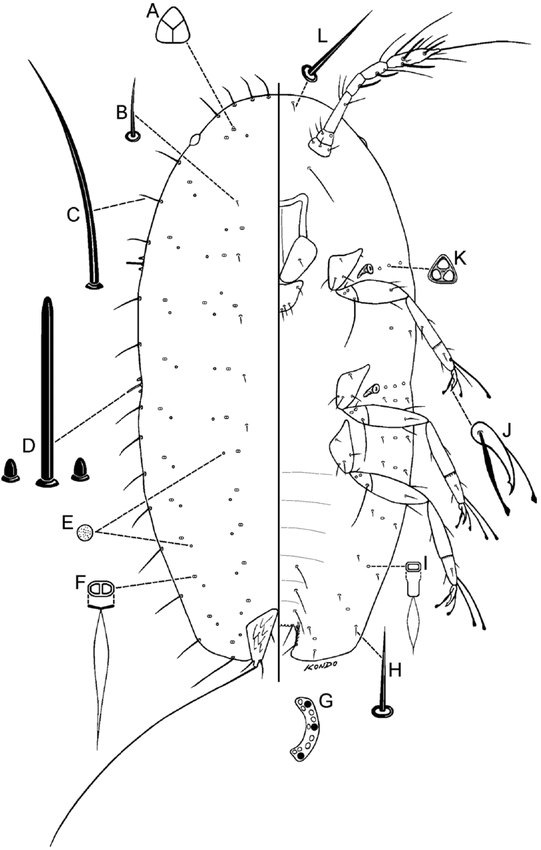
Pseudocribrolecanium andersoni (Newstead), first-instar nymph. A, trilocular pore; B, dorsal seta; C, marginal seta; D, stigmatic setae; E, simple pore; F, dorsal microduct; G, anal ring (right half); H, ventral seta; I, ventral microduct; J, claw; K, spiracular disc-pore; L, ventral submarginal cephalic seta.

**Pseudocribrolecanium andersoni(Newstead), comb. nov.**
Figures 1, 2
The White Powdery Scale
Akermes andersoni Newstead, 1917: 347; Almeida 1969: 135; De Lotto, 1965: 178; Hall 1937: 122
Lecanium andersoni Newstead; Green & Laing 1924: 419
Cribrolecanium andersoni (Newstead); De Lotto 1968: 83; Brink & Bruwer 1989: 9; Hodgson 1969: 10
Parakermes andersoni (Newstead); Fonseca, 1973: 247

### Material studied

#### Lectotype

In order to preserve stability and the nomenclatural status of this species, a lectotype is here designated from the syntypes of this species as follows: “Lecanium andersoni”, Kenya (as British East Africa), 1(1): collecting date not given (according to the Type description, the date should be i.1914), coll. T.J. Anderson, ex orange, No. 18, 14/252, labeled cotype, B.M. 1945, 121 (BMNH).

#### Paralectotypes

Kenya (as B.E. Africa), 1(5): Kabete, i.1914, ex orange, coll. T.J. Anderson, B.M. 1945, 121, (BMNH); 1(3): collecting date not given, slide mounted from Newstead's collection (the collector T.J. Anderson does not appear on the label), ex citrus, labeled part of type material, B.M. 1940, No. 180 (BMNH); 1(4): Limoru, i.1914, coll. T.J. Anderson, ex orange, No. 14/252, No. 18, labeled cotype, Reg. 1916.30 (BMNH); 1(5: 2 adult ♀♀ + 1 third-instar nymph + 2 Diaspididae): Limoru, i.1914, coll. T.J. Anderson, ex orange, No. 14/252, No. 18, labeled cotype, 1916-30 (BMNH); 1(3 first-instar nymphs): Limoru, i.1914, coll. T.J. Anderson, ex orange, No. 14/252, No. 18, labeled “Type larvae”, 1916–30 (BMNH).

#### Non-type material

Ghana, 1(1): Tofo, 21.v.1976, coll. not given, ex cacao, COPR, C.I.E.A. 9866 (BMNH); Kenya, 1(2): Westlands, 23.viii.1973, coll. not given, ex Ficus, No. 2108, G.W. 0100, C.I.E.A. 6593 (BMNH); 1(1): Juja, 23.ix.1978, coll. T.C. Griesbach, 2175, C.I.E.A.10823 (BMNH); 1(2): Nairobi, 8.i.1982, coll. Alice, ex passionfruit, No. 60/83 TC 2219, CIEA 13866 (BMNH); Tanzania (as German East Africa), 19(19): 2.iii.1918, coll. Lamborn, S.W.R., host not given, AL-133-99, slide mounted from dry material (BMNH); 11(11: first-instar nymphs): 2.iii.1918, coll. S.W.R. Lamborn, det. Newstead, host not given, slide-mounted from BMNH dry material, AL-133-99c–m (AUCC); Uganda, 1(2: 1 adult ♀+ 1 adult ♂): Kampala, 25.viii.1919, coll. C.C. Gowdey, ex Citrus nobilis, B.M. Reg. No. 1919.253 (BMNH). Zambia (as Northern Rhodesia), 1(2): Nkana, 6.vi.1948, coll. J. Fourfooz, det. W.J. Hall, host not given, I.I.E. 1521/1969, BM1958-758 (BMNH). Zimbabwe (Southern Rhodesia), 2(2): Mazoe, Umba, 24.iii.1935, coll. M.C. Mossop, ex grape fruit leaves, B.M. 1958-229, No. 822 (BMNH); 1(1): Mazoe, Umba, 26.v.1935, coll. Mossop per W.J. Hall, via Green, det. F. Laing, ex grape fruit leaves, No. 822 (BMNH); 1(1): Harare (as Salisbury), 27.ix.1962, coll. not given, ex citrus, det. C.J. Hodgson, (815), Acc. No. 9877, BM1967-758 (BMNH). Country not given (probably Zimbabwe), 1(3): Imbija, 10.xi.1938, Pres. Dr. W.J. Hall, ex grapefruit, B.M. 1958.229 (BMNH).

### Description

Adult female (Fig. 1)

#### Unmounted material

Insects completely covered with rather dense, dusky-white, mealy secretion. Color on removal of secretion, rich dark piceous or very dark castaneous, shining; younger specimens varying from reddish brown to dusky buff. Form irregularly oval, asymmetrical, more or less narrowed in front; sometimes broadly ovate or subcircular. Flat or very low convex, with faint median keel in abdominal region; sides well within margin. Derm densely sclerotized, especially towards margins; with innumerable minute, translucent poreless cells (Newstead 1917).

#### Mounted material

Body outline oval, pyriform to slightly asymmetrical; body 2.1–3.8 mm long, 1.4–3.2 mm wide (n=48).

#### Dorsum

Derm often with areolations, particularly expressed submarginally (Fig. 1B). Dorsal setae (Fig. 1E) sharply spinose or conical, each 5–9 μm long. Simple pores (Fig. 1G) about 1.8 μm wide, scattered on dorsum. Dorsal microducts (Fig. 1F) each about 1.8 μm wide, scattered on dorsum. Translucent stigmatic furrows present as in diagnosis, usually with an additional translucent lateral furrow on head and two on abdominal region. Cribriform plates (Fig. 1A) oval to elongate oval, occurring either submarginally or submarginally and submedially, totaling 12–22; varying in size, plates closer to margin smallest, each about 10–80 μm wide, innermost plates largest, each 100–180 μm wide. Preopercular pores present in a linear group of about 10–40 pores anterior to anal plates, often displaced anteriorly onto the thoracic and head region with pores completely absent just anterior to anal plates (as reported by Almeida 1969), or form a long line of pores extending from area just anterior to anal plates up to head region. A sclerotic area often present around anal plates. Anal plates (Fig. 1H) together inverted pyriform or quadrate, with round angles, located at about 1/6 of body length from posterior margin, each plate 117–121 μm long, 53–58 μm wide; anterolateral margin 62–70 μm long, posterolateral margin 98–107 μm long, with 3 or 4 setae on dorsal surface, plus 2 pairs of fringe setae and about 2 ventral subapical setae. Anal ring (Fig. 1J) as in diagnosis. Eyes present, represented by clear subcircular areas located on dorsal submargin (see arrow on Fig. 1).

#### Margin

A strongly sclerotized narrow band present around margins (Fig. 1C) in older specimens. Marginal setae (Fig. 1D) flagellate, each 46–115 μm long, arranged in a single row, numerous, with 24–28 on each side between anterior and posterior stigmatic areas. Stigmatic clefts shallow, with 3, rarely less or up to 5 stigmatic setae (Fig. 1C), each seta bluntly spinose; 5–10 μm long, often with one seta much longer than others, 10–15 long.

#### Venter

Ventral setae (Fig. 1L) slender, straight or slightly bent, each 7–16 μm long. Ventral microducts (Fig. 1M) scattered evenly on venter, small, each about 1.5 μm wide. Mouthparts centered or less often displaced to one side; clypeolabral shield 145–156 μm wide. Pregenital disc-pores (Fig. 1I) each 3.6–4.4 μm wide, with 6–11 loculi, mostly 10-locular, present around vulvar area and anteriorly across all abdominal segments, becoming gradually scarce on anterior segments. Spiracular disc-pores (Fig. 1N) each 3–4 μm wide, with 3–5 loculi, in a single line extending laterally from each spiracle to body margin. Antennae (Fig. 1O) rather short, each 111–145 μm long, segmentation indistinct, with 2–4 discernible segments; with fleshy setae present on last segment only and often apically knobbed. With 2 pairs of interantennal setae. Legs (Fig. 1K) each 23–44 μm long (total length); tarsal digitules not detected; claw digitules, slender, setose. Anterior spiracular peritremes each 31–34 μm wide, posterior peritremes each 37–41 μm wide.

#### Morphological variation

Two specimens from Kenya labeled as A. andersoni collected on Ficus sp. are strongly asymmetrical and resemble P. colae. Their largest cribriform plates are rather small, each 40–80 μm wide, smallest cribriform plates each 10–40 μm wide. The cribriform plates in these species are arranged mostly in a single marginal row although there are places with definitely an inner and outer row as in the rest of the material of P. andersoni. The total number of cribriform plates in the Kenyan specimens are 14 and 16 respectively and fall also in the range of P. andersoni. Whether the specimens collected on Ficus sp. should belong in a different species needs further study. Measurements of the above specimens were excluded from the description.

First-instar nymph (Fig. 2)

#### Unmounted material

Not seen.

#### Mounted material

Body outline elongate oval, anterior end rounded; body 431–485 μm long, 189–226 μm wide (n=14).

#### Dorsum

Dorsal setae (Fig. 2B) short, each 4–5 μm long, distributed as in diagnosis. A trilocular pore (Fig. 2A) present on each side of head region near margin. Dorsal microducts (Fig. 2F) each 2.7–3.6 μm wide, distributed as in diagnosis. Simple pores (Fig. 2E) each about 1.8 μm wide and closely associated with a microduct. Anal plates each 48–50 μm long, 22–24 μm wide; anal plate setae and anal ring (Fig. 2G) as in diagnosis.

#### Margin

Marginal setae (Fig. 2C) slender, each 13–33 μm long, those on anterior and posterior ends longest; number and distribution as in diagnosis. Each stigmatic area with a group of 3 stigmatic setae (Fig. 2D), well differentiated from marginal setae; each median seta bluntly spinose, 15–17 μm long; lateral setae bulbous, each 1.8–2.7 μm long.

#### Venter

Submedian abdominal setae in pairs on posterior 3 segments, each seta 13–22 μm long, those on last abdominal segments longest. Submarginal setae (Fig. 2H & L) distributed as in diagnosis. Ventral microducts (Fig. 2I) each about 1.5 μm wide, distributed as in diagnosis. Spiracular disc-pores (Fig. 2K) each 2–3 μm wide, with 3 loculi; with 3 or 4 pores present in a line from each spiracle to margin. Clypeolabral shield 67–71 μm wide. Trochanter + femur of each leg: 68–74 μm long, tibia + tarsus 65–68 μm long, claw (Fig. 2J) 13–15 μm long. Antennae 6-segmented, each 130–138 μm long.

### **Biology (from** Brink 1998)

The white powdery scale is a polyphagous insect known as a pest of citrus. The adult female is completely covered with a dense white powdery secretion, which also spreads over the surrounding parts of the host plant giving infested leaves an almost uniform white powdery appearance. P. andersoni feeds on leaves and young shoots, but does not feed on the fruit and branches. The honeydew serves as a substrate for the saprophytic sooty mold fungi of the genus Capnodium. In South Africa, the insect is found throughout the year, where it appears to have three field generations per year. Various natural enemies of P. andersoni are known, including the hymenopterous parasitoids Coccophagus philippiae (Silvestri), C. pulvinariae Compere, C. lycimnia (Walker), Coccophagus sp. (Aphelinidae), Metaphycus spp., Neastymachus dispar Prinsloo (Encyrtidae), Tetrastichus sp. (Eulophidae), and chrysopid larvae (Neuroptera). P. andersoni was considered parthenogenetic by Brink (1998) and Brink & Brewer (1989). However, Almeida (1969) described the adult male and an adult male was observed in the present study (see Material studied).

### Diagnostic features

The adult female of P. andersoni (Newstead) can be easily separated from P. colae by the following combination of characters: (i) presence of both large and small cribriform plates on P. andersoni (all small on P. colae ), (ii) difference in number of cribriform plates, with 10–22 in P. andersoni, but only 0–9 on P. colae, (iii) body shape, which is oval to only slightly asymmetrical in P. andersoni but highly asymmetrical in P. colae, and (iv) the presence of obvious simple pores (not detected on P. colae). Furthermore, the first-instar nymphs of P. andersoni have (v) bluntly spinose stigmatic seta whilst they are clavate on P. colae, and (vi) the head has a round contour whereas it is tapering in P. colae.

### Distribution

Afrotropical Region: Angola, Cameroon, Ghana, Kenya, Mozambique, South Africa, Swaziland, Uganda, Zambia, Zimbabwe, Mauritius (Brink 1998; De Lotto 1965; Grové 2001; Hall 1937; Hodgson 1969; Matile-Ferrero & Nonveiller 1984).

### Host plants

Anacardiaceae: Mangifera indica; Araliaceae: Schefflera sp.; Boraginaceae: Ehretia silvatica; Lauraceae: Persea americana; Loganiaceae:Anthocleista grandiflora, Strychnos madagascariensis; Meliaceae: Toona ciliata; Moraceae: Ficus benjamina, F. elastica, F. sur, F. verrucocarpa; Myrtaceae: Callistemon sp., Psidium guajava; Passifloraceae: Passiflora edulis; Rubiaceae: Psychotria zombamontana, Coffea canephora; Rutaceae: Citrus paradisi, C. reticulata, Citrus spp.; Sterculiaceae: Theobroma cacao; Strelitziaceae: Strelitzia nicolai (Almeida 1969; Ben-Dov 1993; Brink 1992a, b, 1998; De Lotto 1965; Grové 2001; Hall 1937; Hodgson 1969; Matile-Ferrero & Nonveiller 1984).

**Figure 3.**
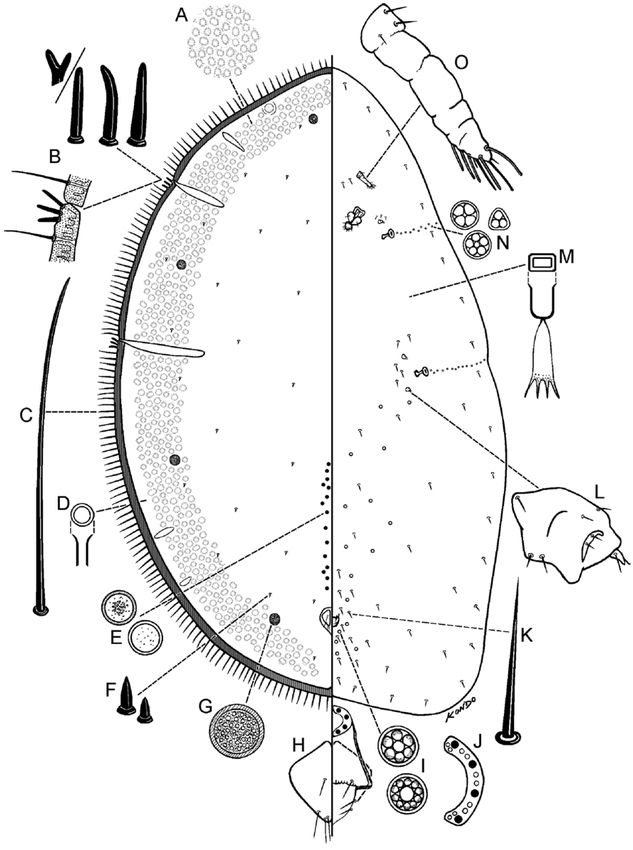
Pseudocribrolecanium colae (Green & Laing), adult female (drawn from type material). A, enlargement of dorso-submarginal derm; B, stigmatic setae (top) and enlargement of sclerotized marginal band with marginal and stigmatic setae (below); C, marginal seta; D, dorsal microduct; E, preopercular pores; F, dorsal setae; G, cribriform plate; H, anal plate; I, pregenital disc-pores; J, anal ring (right half); K, ventral seta; L, reduced leg; M, ventral microduct; N, spiracular disc-pores; O, antenna.

**Figure 4.**
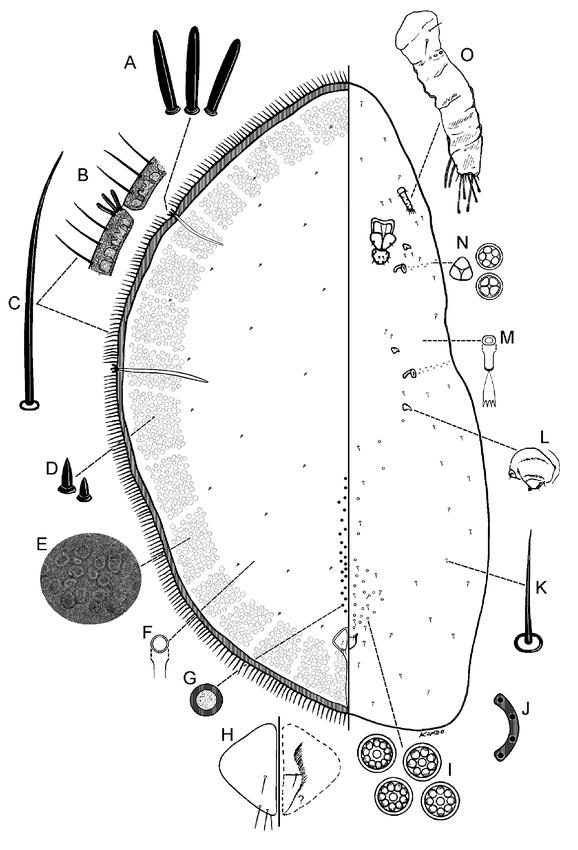
Pseudocribrolecanium colae (Green & Laing), adult female (drawn from specimen collected in Ghana). A, stigmatic setae; B, enlargement of sclerotized marginal band with marginal and stigmatic setae; C, marginal seta; D, dorsal setae; E, enlargement of dorso-submarginal derm; F, dorsal microduct; G, preopercular pore; H, anal plate; I, pregenital disc-pores; J, anal ring (right half), wax pores not illustrated; K, ventral seta; L, reduced leg; M, ventral microduct; N, spiracular disc-pores; O, antenna.

**Figure 5.**
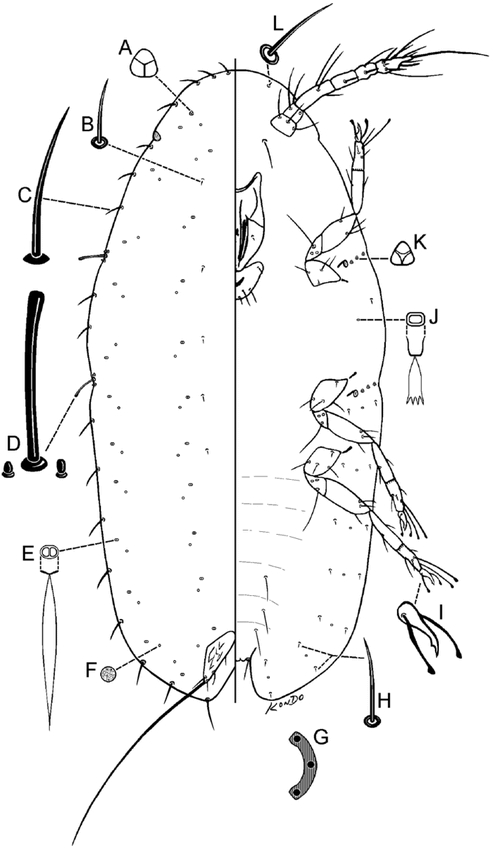
Pseudocribrolecanium colae (Green & Laing), first-instar nymph (drawn from specimen collected in Ghana). A, trilocular pore; B, dorsal seta; C, marginal seta; D, stigmatic setae; E, dorsal microduct; F, simple pore; G, anal ring (right half); H, ventral seta; I, claw; J, ventral microduct; K, spiracular disc-pore; L, ventral submarginal cephalic seta.

**Pseudocribrolecanium colae (Green & Laing), comb. nov**
Figures 3, 4, 5
Lecanium (Akermes) colae Green & Laing, 1924: 419
Akermes colae Green & Laing, Ben-Dov 1993: 5

### Material studied

#### Lectotype

In order to preserve the stability and nomenclatural status of this species, a lectotype of this species is here designated from the syntypes of this species as follows: “Lecanium (Akermes) colae”, Ghana, 1(1): Gold Coast, Aburi, 24.i.1922, coll. W.H. Patterson, det. Laing, ex Cola acuminata, No. 610 (BMNH).

#### Paralectotypes

GHANA, same data as lectotype, mixed slide, 1(6: 1 hymenopteran larva + 4 adult ♀♀ Parasaissetia nigra (Nietner) + 1 adult ♀P. colae ): paralectotype conspecific with lectotype clearly indicated on label: longest of all specimens on slide ca. 3.5 mm (at greatest length) (BMNH).

#### Other material studied

Sierra Leone, 4(8: 3 adult ♀♀+ 5 second-instar ♂♂): Freetown, Sama, 25.xi.1924, coll. E. Hargreaves, ex. avocado pear, No 1268 (BMNH). Ghana, 2(2: 1 adult ♀+ 1 first-instar nymph): Eastern Region, Bunso Arboretum, 06°16′02″ N, 00°27′44″ W, 19.vi.2005, coll. G.E. Morse & T. Kondo, ex Theobroma cacao, underside leaf along vein (BMNH).

#### Note

The collection data of the lectotype and paralectotype herein designated matches well the data given in the original description by Green & Laing (1924). However, the paralectotype slide is composed of a mixture of specimens of which only one specimen is conspecific with the lectotype. The remaining specimens include four adult females of the coccid Parasaissetia nigra (Nietner) and one hymenopteran larva (probably a parasitoid). It is common to find one or two, and even more species together on one host plant. The mixing of species on this one slide probably occurred because the adult females of the two coccid species resemble each other superficially in life, e.g., P. nigra and P. colae both become heavily sclerotized at maturity and their body shape is somewhat similar being pointed at both extremities.

### Description

Adult female (Figs 3 & 4)

#### Unmounted material

Morphology not recorded.

#### Mounted material

Body outline asymmetrical, head acuminate; body 2.0–2.2 mm long, 3.3–3.7 mm wide (n=4).

#### Dorsum

Derm with numerous areolations in a broad submarginal band; area of areolation often narrower or less pronounced on one side of body (enlargement of dorsal derm shown in Figs 3A, 4E). Areolated submarginal band often with lighter subcircular patches of less sclerotized areas present at regular intervals. Dorsal setae (Figs 3F, 4D) spinose, short, each 2–3 μm long. Simple pores not detected. Dorsal microducts (Figs 3D, 4F) small, each about 2 μm long, inner ductule not detected, scattered throughout dorsum. Cribriform plates (Fig. 3F) present or absent (Fig. 4), if present, subcircular, each 15–45 μm wide, located submarginally; total 7–9: with 1–3 on head region, 1 between stigmatic areas, and 2 between posterior stigmatic area and body apex. Translucent stigmatic furrows present as in diagnosis, usually with an additional submarginal lateral furrow on head and 2 on abdominal region. Preopercular pores (Figs 3G, 4G) each 4.5–8.0 μm wide, in 1 or rarely 2 groups totaling 12–25 pores in a straight longitudinal line 1 or 2 pores wide anterior to anal plates; pore line extending up to area about half way between anal plates and posterior spiracular furrows; pores absent from area immediately anterior to anal plates. Anal plates (Figs 3H, 4H) together quadrate, each with round angles; located about 1/7 of body length from posterior margin; each plate 43–45 μm long, 25–26 μm wide; anterolateral margin 32–33 μm long, posterolateral margin 37–39 μm long; each with 4 setae on dorsal surface, plus 1 pair of fringe setae and about 2 ventral subapical setae. Anal ring (Figs 3J, 4J) as in diagnosis. Eyes present, represented by clear subcircular areas on dorsal submargin.

#### Margin

Margins becoming heavily sclerotized in a narrow band around entire body on older specimens. Marginal setae (Figs 3C, 4C) flagellate, each 62–72 μm long, arranged in a single row, numerous, with 23–31 on each side between anterior and posterior stigmatic areas. Stigmatic clefts very shallow or absent; each with 3, rarely 4 stigmatic setae (Figs 3B, 4A&B), often absent or broken off in some stigmatic areas; each setae sharply spinose, rarely bifurcate, 7.5–20 μm long; median seta longest, or all subequal in length.

#### Venter

Ventral setae (Figs 3K, 4K) slender, straight or slightly bent, each 8–18 μm long, sparsely distributed submarginally, near appendages, and on mid areas of venter. Ventral microducts (Fig. 3M, 4M) sparse, scattered evenly on venter, small, each about 1.5 μm wide. Mouthparts displaced to one side; clypeolabral shield 54–55 μm wide. Pregenital disc-pores (Fig. 3I, 4I) each 3.6–4.5 μm wide, with 5–10 (mostly 10) loculi, present around vulvar region and sparsely distributed on mid areas of abdomen. Spiracular pores (Figs 3N, 4N) each 2.7–3.6 μm wide, with 3–5 loculi, present in an irregular line 1 or 2 pores wide extending laterally from each spiracle to body margin. Antennae (Figs 3O, 4O) small, each 38–44 μm long, segmentation indistinct, with about 3–5 discernible segments; setal distribution as in diagnosis. With 2 pairs of interantennal setae. Legs (Figs 3L, 4L) greatly reduced, each 8–16 μm long (total length), all segments fused; claws rarely visible; claw and tarsal digitules not discernible. Spiracles rather small; anterior spiracular peritremes each 25–27 μm wide, posterior peritremes each 30–32 μm wide.

#### Morphological variation

A specimen recently collected in Ghana (Fig. 4) has fewer translucent furrows, no lighter patches around submargins and no cribriform plates. The degree of sclerotization appears to be highly variable in P. colae. Thus the main difference between the specimen from Ghana and those from the type series is the presence or absence of cribriform plates. More material is needed to determine if the specimen without cribriform plates is part of the morphological variation of A. colae or a separate closely-related species.

First-instar nymph (Fig. 5)

#### Unmounted material

Morphology not recorded.

#### Mounted material

Body outline elongate oval, tapering towards head; body 575 μm long, 275 μm wide (n=1).

#### Dorsum

Dorsal setae (Fig. 5B) short, each 4–5 μm long, distributed as in diagnosis. A trilocular pore (Fig. 5A) present on each side of head region near margin. Dorsal microducts (Fig. 2E) each about 2.0 μm wide, distributed as in diagnosis. Simple pores (Fig. 5F) each about 1.5 μm wide, each present near a microduct. Anal plates each 48 μm long, 18 μm wide; setae as in diagnosis. Anal ring (Fig. 5G) as in diagnosis.

#### Margin

Marginal setae (Fig. 5C) slender, each 13–41 μm long, longest towards posterior apex; number and distribution as in diagnosis. Stigmatic setae (Fig. 5D): median seta longest, clavate, each 22–25 μm long; lateral setae bulbous, short, each 2–4 μm long.

#### Venter

Abdomen with pairs of setae on posterior 3 segments, each 22–25 μm long, those on posterior abdominal segment longest. Submarginal setae (Fig. 5H & L) distributed as in diagnosis. Ventral microducts (Fig. 5J) each 1.5–2.0 μm wide, distributed as in diagnosis. Spiracular disc-pores (Fig. 5K) each about 2 μm wide, with 3 loculi; present in a line of 3 pores from each spiracle to margin. Clypeolabral shield 55 μm wide. Legs well-developed, each trochanter + femur 60–73 μm long, tibia + tarsus 65–70 μm long, claw (Fig. 5I) 15–16 μm long; prothoracic legs shortest. Antennae 6-segmented, each 133–138 μm long.

### Biology

No information on the biology of P. colae has been published. The single specimen of the form without cribriform plates collected recently in Ghana was on the under surface of a leaf of Theobroma cacao alongside the leaf vein. The presence of second-instar males (see Material studied) suggests that the species reproduces sexually.

### Diagnostic features

See diagnostic features under P. andersoni.

### Distribution

Afrotropical Region: Ghana, Sierra Leone.

### Host plants

Sterculiaceae: Cola acuminata, Theobroma cacao. Lauraceae: Persea americana.

## Discussion

Pseudocribrolecanium appears similar to Cribrolecanium Green (Myzolecaniinae), with which it shares dorsal cribriform plates and highly reduced appendages. However, this resemblance is superficial. Cribriform plates are also present on some other coccids. Hodgson (1994) describes the following genera as having cribriform plates: Eutaxia Green, Hemilecanium Newstead and Stictolecanium Cockerell (Coccinae: Saissetiini); and Halococcus Takahashi and Myzolecanium kibarae Beccari (Myzolecaniinae: Myzolecaniini). Furthermore, reduction of appendages is observed frequently on many members of the Coccidae, e.g. the Myzolecaniinae, Coccinae (Coccini & Paralecaniini in part), Antandroya spp., Lecanochiton spp., Neolecanochiton grevillea Hempel, Paracardiococcus actinodaphnis Takahashi, Platinglisia noacki Cockerell, Pseudokermes spp., Schizochlamidia mexicana Cockerell & Parrott (Cardiococcinae), Cissococcus fulleri Cockerell (Cissococcinae), Cyphococcus caesalpiniae Laing (Cyphococcinae), Scythia craniumequinum Kiritshenko (Eriopeltinae) and Physokermes spp. (Eulecaniinae).

Cribrolecanium possesses the following features that separate it from Pseudocribrolecanium (character states on Pseudocribrolecanium in brackets): (i) the presence of a membranous bag-like structure which contains the spiracular pores (see Hodgson 1995) (spiracular disc-pores not within a bag-like structure), (ii) the restriction of pregenital disc-pores to immediately around the vulvar region (spreading anteriorly), (iii) the lack of spiracular setae (3–5), (iv) complete absence of preopercular pores (present), (v) presence of small groups of dorsal microducts on area anterior to anal plates (absent), (vi) antennal length less than the width or only slightly longer, never twice its width (length of antennae 3–4 times its width), and (vii) an anal ring with 16–18 setae (8 in Pseudocribrolecanium ). The two genera also differ greatly at the ecological level, with Cribrolecanium being found on the roots and in hollow branches of its host, whereas Pseudocribrolecanium feeds on the leaves.

Pseudocribrolecanium shares important features with the Paralecaniini (Coccinae), particularly with species of the genus Paralecanium Cockerell. These shared features are: (i) the position of the eyes on the dorsum away from the margin, (ii) a marginal sclerotized band, (iii) translucent furrows, and (iv) numerous marginal setae. However, in Paralecanium, as well as other genera in the Paralecaniini, the multilocular disc-pores are restricted to immediately anterior to the genital opening and the stigmatic clefts are usually quite distinct (Hodgson 1994). In Pseudocribrolecanium, the multilocular disc-pores are found also on some anterior abdominal segments, and the stigmatic cleft is poorly developed or lacking. In addition, the morphology of the first-instar nymphs does not support a close relation with the Paralecaniini. Very few studies have described or illustrated the first-instar nymphs of the Paralecaniini, but those species which have been described (e.g., Austrolecanium cappari (Froggatt), A. sassafras Gullan & Hodgson, Maacoccus cinnamomicolus (Takahashi), Paralecanium paradeniyense Green, P. planum (Green), and Xenolecanium takahashii Kondo) share many important features, i.e., a seta next to each mesothoracic and metathoracic coxa, six pairs of ventral submedian setae, and stigmatic setae positioned submarginally on either side of a deep stigmatic cleft (Gullan & Hodgson 1998, Hodgson and Martin 2001, Kondo et al. 2005). However, on the first-instar nymphs of Pseudocribrolecanium, the stigmatic clefts are very shallow, the stigmatic setae are positioned along the margins and not on the sides of a deep stigmatic cleft, and no seta is found next to either the meso-and metathoracic coxae, suggesting that Pseudocribrolecanium is not closely related to the Paralecaniini despite the similarities shared by the adult females. Further studies using additional material, molecular techniques and adult males, may help elucidate the phylogenetic position of Pseudocribrolecanium.
